# Proteasome, but Not Autophagy, Disruption Results in Severe Eye and Wing Dysmorphia: A Subunit- and Regulator-Dependent Process in *Drosophila*

**DOI:** 10.1371/journal.pone.0080530

**Published:** 2013-11-25

**Authors:** Panagiotis D. Velentzas, Athanassios D. Velentzas, Asimina D. Pantazi, Vassiliki E. Mpakou, Christos G. Zervas, Issidora S. Papassideri, Dimitrios J. Stravopodis

**Affiliations:** 1 Department of Cell Biology and Biophysics, Faculty of Biology, University of Athens, Athens, Greece; 2 Genetics Division, Center of Basic Research II, Biomedical Research Foundation, Academy of Athens, Athens, Greece; Foundation for Biomedical Research Academy of Athens, Greece

## Abstract

Proteasome-dependent and autophagy-mediated degradation of eukaryotic cellular proteins represent the two major proteostatic mechanisms that are critically implicated in a number of signaling pathways and cellular processes. Deregulation of functions engaged in protein elimination frequently leads to development of morbid states and diseases. In this context, and through the utilization of GAL4/UAS genetic tool, we herein examined the *in vivo* contribution of proteasome and autophagy systems in *Drosophila* eye and wing morphogenesis. By exploiting the ability of GAL4-ninaE. GMR and P{GawB}Bx^MS1096^ genetic drivers to be strongly and preferentially expressed in the eye and wing discs, respectively, we proved that proteasomal integrity and ubiquitination proficiency essentially control fly’s eye and wing development. Indeed, subunit- and regulator-specific patterns of severe organ dysmorphia were obtained after the RNAi-induced downregulation of critical proteasome components (Rpn1, Rpn2, α5, β5 and β6) or distinct protein-ubiquitin conjugators (UbcD6, but not UbcD1 and UbcD4). Proteasome deficient eyes presented with either rough phenotypes or strongly dysmorphic shapes, while transgenic mutant wings were severely folded and carried blistered structures together with loss of vein differentiation. Moreover, transgenic fly eyes overexpressing the UBP2-yeast deubiquitinase enzyme were characterized by an eyeless-like phenotype. Therefore, the proteasome/ubiquitin proteolytic activities are undoubtedly required for the normal course of eye and wing development. In contrast, the RNAi-mediated downregulation of critical Atg (1, 4, 7, 9 and 18) autophagic proteins revealed their non-essential, or redundant, functional roles in *Drosophila* eye and wing formation under physiological growth conditions, since their reduced expression levels could only marginally disturb wing’s, but not eye’s, morphogenetic organization and architecture. However, Atg9 proved indispensable for the maintenance of structural integrity of adult wings in aged flies. *In toto*, our findings clearly demonstrate the gene-specific fundamental contribution of proteasome, but not autophagy, in invertebrate eye and wing organ development.

## Introduction

*Drosophila melanogaster* is a genetically powerful and biologically invaluable animal model system, which provides all the cellular and molecular tools to reliably examine the effects of proteasome and autophagy -targeted- disruption in organ development. Eye and wing morphogenesis in *Drosophila* are two of the most recognized developmental processes, both playing critical roles in fly’s patho-physiology. *Drosophila* compound eye is considered as one of the most precise and highly ordered morphogenetic pattern in the living world, as well as a favourite system of geneticists in studies of developmental mechanisms and analysis of gene expression [1], [2]. Therefore, *Drosophila* eye undoubtedly represents an ideal developmental platform for forward genetic screens and targeted inhibition of pivotal cellular mechanisms. *Drosophila* wing development represents another classical ontogenetic model, mostly used for studying the genetic control of tissue size, shape and patterning [3], [4].

The ubiquitin/proteasome machinery in eukaryotes regulates a number of critical cellular processes, such as signal transduction, cell cycle progression, transcriptional activation, inflammation and apoptosis [5]. Proteasome particles exist in abundance, representing almost 1% of total cellular protein content, in both nucleus and cytoplasm of all eukaryotic cells and display high levels of specificity towards their numerous cognate substrates. Ubiquitin/proteasome-dependent protein degradation is usually carried out in two successive and distinct steps. Initially, selected proteins, targeted for destruction, are tagged by covalent addition of ubiquitin molecules, through the E1–E3 ubiquitin-conjugation system, and subsequently they are recognized and digested by the multi-functional 26S proteasome machinery [6]. The 26S proteasome, an ATP-dependent protease complex, is structured by two multi-subunit sub-complexes: a 20S catalytic core, which is organized into 4 stacked rings, specified as β (“beta”) inner rings and α (“alpha”) outer rings, and made up of 7 subunits, and two 19S proteasome activator regulatory caps [7]. 19S caps are responsible for the recognition of ubiquitinated proteins, while they also posses ATPase activity mainly required for unfolding and sequential transferring of the de-ubiquitinated target proteins into the interior of proteasome core particle. Protein degradation is implemented by three proteolytically active β subunits per β ring, characterized by different hydrolytic activities and substrate specificities [8].

When poly-ubiquitinated protein aggregates accumulate in the cells during stress, aging or disease, autophagy is often induced for their clearance [9]–[11]. Autophagy is an evolutionarily conserved physiological process that primarily acts as a cytoprotective mechanism in order to maintain nutrient and energy homeostasis under cellular stress. However, a function for autophagy in cell death has been also documented and therefore autophagy is often called “type II Programmed Cell Death (PCD)”. Autophagic cell death plays a significant role in development, where several morphogenetic programs require massive cell elimination. Moreover, autophagy is also involved in physiology, lifespan and a wide range of diseases, including cancer and neurodegeneration [12]. In mechanistic terms, the most prominent feature of autophagy is the accumulation of double-membrane vacuoles in the cytoplasm, widely known as autophagosomes, the formation of which depends on proteins encoded by the *Atg* genes [12].

To these directions, in the present study, we examined the developmental effects of organ-specific and genetically-mediated disruption of proteasome or autophagy integrity in *Drosophila* eye and wing morphogenesis, via a GAL4/UAS-based overexpression of conditionally defective proteasome subunits or the UBP2 yeast ubiquitin protease. Moreover, an RNAi-induced downregulation of critical components controlling each protein-degradation machinery activities was also employed in the same organs. We found that, unlike autophagy, proteasome impairment results in severe eye and wing dysmorphia, thus indicating proteolytic-system’s differential contribution in fly organ development. A subunit- and regulator-dependent mode of action was also underscored by the distinct mutated phenotypes obtained. Interestingly, the RNAi-mediated downregulation of Atg9 autophagic protein proved to be tightly associated with an age-dependent pathogenic phenotype of broken wings.

## Materials and Methods

### Drosophila Melanogaster Stocks and Culturing Conditions

The following *Drosophila melanogaster* fly strains were used: P{w[+mC] = GAL4-ninaE.GMR}12 (BL: 1104), w [1118]; P{w[+mW.hs] = GawB}Bx [MS1096] (BL: 8860), y[*] w[*]; P{w[+mC] = UAS-2xEGFP}AH2 (BL: 6874), w [1118]; P{w[+mC] = UAS-Prosbeta2 [1]}1B (BL: 6785), w [1118]; P{w[+mC] = UAS-Pros26 [1].B}2B (BL: 6786), w [1118]; P{w[+mC] = UAS-Pros26 [1].B}2B; P{UAS-Prosbeta2 [1]}1B (BL: 6787), w[*]; P{w[+mC] = UAS-UBP2.D}2/CyO (BL: 9907), (all obtained from Bloomington *Drosophila* Stock Center, Indiana University, Bloomington {BL}, Indiana, USA), UAS-Rpn1_RNAi (TID: 25549), UAS-Rpn2_RNAi (TID: 44135), UAS-Rpn6_RNAi (TID: 18022), UAS-alpha5_RNAi (TID: 16105), UAS-dbeta5_RNAi (Transformant ID: 38659), UAS-beta6_RNAi (TID: 34801), UAS-UbcD1_RNAi (TID: 26011), UAS-UbcD4_RNAi (TID: 35873), UAS-UbcD6_RNAi (TID: 23229), UAS-Atg1_RNAi (TID: 16133), UAS-Atg4_RNAi (TID: 34843), UAS-Atg7_RNAi (TID: 45558), UAS-Atg9_RNAi (TID: 10045) and UAS-Atg18_RNAi (TID: 22643) (all obtained from Vienna *Drosophila* RNAi Center {VDRC}, Vienna, Austria). Fly stocks were maintained at 25°C and fed on standard diet. Flies overexpressing the temperature sensitive (ts), dominant negative β2^ts^, β6^ts^ or both β2^ts^ and β6^ts^ proteasome subunits were raised at a temperature of 29°C.

### Scanning Electron Microscopy (SEM)

The external and surface structural organization of each (single, double or triple transgenic) adult fly eye was examined through a Scanning Electron Microscopy (SEM) approach [13]. Preparation of *Drosophila* tissue for SEM analysis was performed as following: five to ten adult flies were pre-fixed for 2 h, at 4°C, in fresh phosphate-buffered saline (PBS) containing 2.5% glutaraldehyde, pH 7.4. The specimens were subsequently washed twice, for 20 min each time, in phosphate-buffered sucrose (4%). Afterwards, they were post-fixed in 2% osmium tetroxide, at 4°C, for 24 h, and washed again in PBS containing 4% sucrose. Tissue dehydration was attained through a graded ethanol series (70%, 80%, 90%, 95% and 100%). Samples were finally subjected to a critical point drying procedure, attached on aluminum stubs, coated with gold in a sputter-coating apparatus (Tousimis, Rockville, Maryland, USA), for 2 min, and visualized under a Philips SEM 515 Scanning Electron Microscope. For each fly double transgenic (and single or triple transgenic) line, a total of more than 75 eye images, from at least three independent crosses, were thoroughly examined.

### Histology and Light Microscopy

*Drosophila melanogaster* (single, double or triple transgenic) flies were collected, and their heads were removed and bisected with a scalpel to allow complete fixation and infiltration of the embedding media [14]. Adult fly heads were processed for light microscopy as following: they were fixed, overnight, at 4°C, with 2.5% glutaraldehyde in 0.1 M sodium cacodylate buffer. After being rinsed twice with sodium cacodylate, for 20 min each time, fly heads were post-fixed, for 2 h, at 4°C, in 0.1 M sodium cacodylate containing 1% osmium tetroxide. After two washes in sodium cacodylate buffer, for 20 min each time, at 4°C, the specimens were dehydrated through a graded series of ethanol concentrations, infiltrated in propylene oxide and embedded in Epon-Araldite (Fullam Inc., New York, USA). Semi-thin sections were cut on a Sorvall MT-1 microtome, stained with 0.5% toluidine blue and observed under a Nikon Eclipse TE-2000S microscope. Light microscopic images of each adult fly eye were taken after anesthetizing the flies and examining them under a BMS 74958 Stereo-microscope. Photographs of adult fly (single, double or triple transgenic) wings were taken under visible light with a Nikon Eclipse TE-2000S microscope. All the examined wings (at least 60 pairs from three independent crosses) were dissected from 2 or 3 days old flies and mounted directly on glass microscope slides under suitable glass cover slips of 0.13–0.17 mm (#1) thickness (Waldemar Knittel, Braunschweig, Germany). For conducting the aging experiments, male P{GawB}Bx^MS1096^ flies were crossed with Atg9_RNAi females and wing structures of the produced progeny were examined every 10 days for a time period of 10 to 70 days. Flies overexpressing the EGFP reporter protein, either in the eye disc or in the wing disc, were visualized under a Nikon Confocal Laser Scanning Microscope (CLSM), model Digital Eclipse C1 (Nikon, Tokyo, Japan).

## Results

### Targeted Disruption of Ubiquitin/proteasome Signaling Integrity Impairs the Architectural Structure of Drosophila Eye in a Component-dependent Manner

**Figure 1:**
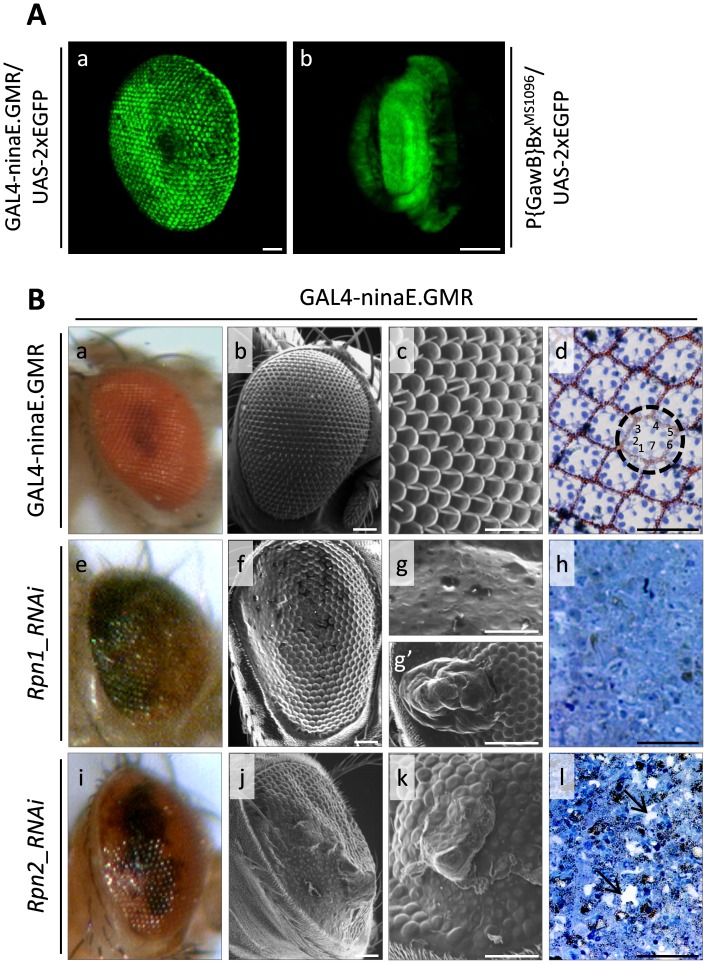
Downregulation of Rpn1 and Rpn2 19S cap proteasome subunits severely affects eye morphogenesis. (A) Confocal Laser Scanning Microscopy (CLSM) images, illustrating the organ-specific and targeted overexpression of EGFP (enhanced green fluorescent protein) reporter protein exclusively in the (a) eye disc of double transgenic flies carrying the GAL4-ninaE.GMR/UAS-2xEGFP strain genotype and (b) dorsal wing disc of double transgenic flies characterized by the P{GawB}Bx^MS1096^/UAS-2xEGFP strain genotype, and therefore reflecting the functional reliability and efficiency of the cell type-dependent genetic scheme employed in the present study. (B) Stereo-microscopical (a, e and i), Optical (d, h and l {semi-thin sections}) and Scanning Electron Microscopy (SEM) (b, c, f, g, g′, j and k) images, demonstrating Drosophila compound eye morphology and structural organization in Rpn1 and Rpn2 functionally deficient cellular environments. (a–d) GAL4-ninaE.GMR single transgenic adult fly compound eyes (dashed circle: ommatidium; 1–7: photoreceptor cells) (utilized as control condition; identical phenotypes are also observed for single transgenic flies grown at 29°C, and for triple transgenic flies overexpressing the β2^ts^ and β6^ts^ -conditional- mutant proteasome components raised at 25°C {data not shown}). (e–h) GAL4-ninaE.GMR/UAS-Rpn1_RNAi double transgenic fly eyes, carrying reduced Rpn1 protein contents. (i–l) GAL4-ninaE.GMR/UAS-Rpn2_RNAi double transgenic fly eyes, characterized by downregulated Rpn2 proteasome subunit expression levels. Arrows: lesion area(s). Scale Bars: 50 µm.

Tissue-specific targeted expression of selected (mutant or heterologous) proteins or RNAi moieties has been, herein, used to disrupt fly’s proteasome activities in different organs. Double transgenic flies were generated through the GAL4/UAS binary genetic system [15], directing the strong overexpression of certain conditionally defective proteasome determinants (or its signalling suppressors) or selected RNAi species, and, thus, targeting the proteasome deregulation and malfunction, preferentially in the eye disc (for control crosses, see Fig. 1Aa). This approach provides a powerful genetic tool to reliably investigate the *in vivo* requirement of proteasome activities throughout the course of *Drosophila* eye development. To this direction, a series of double (or triple) transgenic fly strains carrying, specifically in the eye disc cells, (a) conditionally (temperature sensitive {ts}) defective β2, β6 or β2 and β6 mutant subunits, (b) downregulated (via RNAi technology) Rpn1, Rpn2, Rpn6, α5, β5 and β6 subunits of 26S proteasome, (c) reduced expression (via RNAi technology) of UbcD1, UbcD4 and UbcD6 (E2 ubiquitin conjugating enzymes) proteasome critical regulators and (d) an overexpressed UBP2 yeast ubiquitin protease, were thoroughly examined for all kinds of deformity in the architectural structure and organization of fly eye.

*Drosophila* eye is composed of approximately 750 to 800 identical unit eyes, called ommatidia, organized in an architectonically perfect hexagonal array (Figs. 1Ba–c). Each ommatidium consists of approximately 20 cells. Specifically, 6 rod-like photoreceptor cells (R1–R6) and 2 cone-like photoreceptors (R7 and R8) structure the retina, 6 epithelial cells produce the corneal lens and approximately 6 highly pigmented cells are shared between ommatidia to limit light scatter and participate in chromophore formation (Fig. 1Bd). Finally, an interommatidial mechanosensory bristle is also present at alternating ommatidial apexes [16]. These unique structural features are mainly responsible for the plethora of the observed eye phenotypes arising from mutations, or functional deregulations, of certain genetic determinants that critically control ommatidia morphogenesis. In turn, a genotype-phenotype correlation can be easily recognized by external observation of the obtained eye shape and organization without further manipulation.

**Figure 2:**
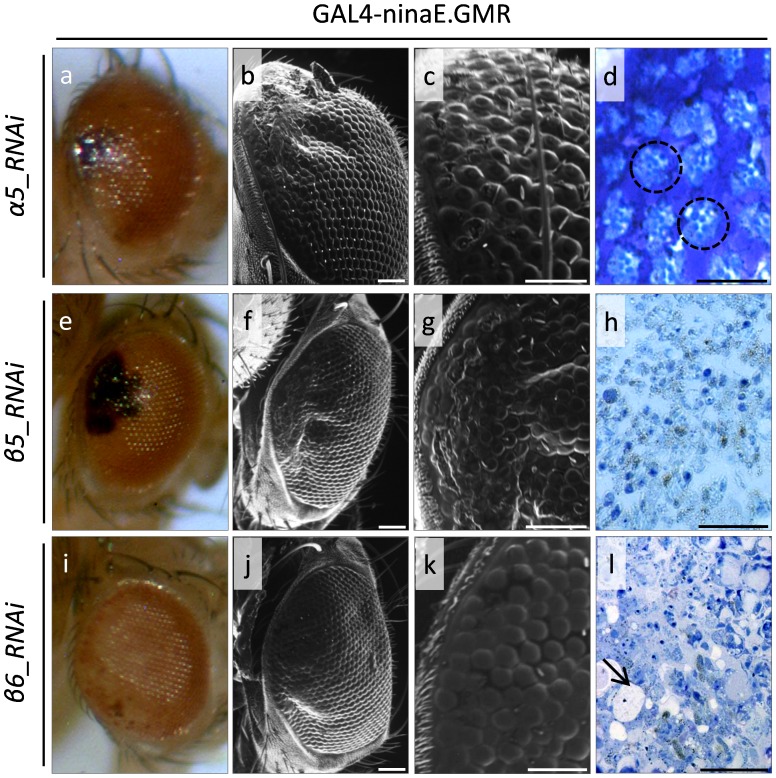
The structural integrity of 20S proteasome core particle is critically implicated in Drosophila eye morphogenesis. Stereo-microscopical (a, e and i), Optical (d, h and l {semi-thin sections}) and Scanning Electron Microscopy (SEM) (b, c, f, g, j and k) images, revealing the damaged architectural pattern of fly eye in 20S proteasome deficient environments (arrow). (a–d) GAL4-ninaE.GMR/UAS-alpha5_RNAi double transgenic fly eyes, carrying reduced α5 protein levels. (e–h) GAL4-ninaE.GMR/UAS-dbeta5_RNAi double transgenic fly eyes, characterized by downregulated β5 proteasome core protein expression levels. (i–l) GAL4-ninaE.GMR/UAS-beta6_RNAi double transgenic flies, producing eyes with decreased β6 cellular contents. Dashed circle: ommatidium. Scale Bars: 50 µm.

We, herein, demonstrate that flies bearing, through the GAL4-ninaE.GMR genetic driver that is strongly and preferentially expressed in the eye disc, downregulated Rpn1, Rpn2, α5, β5 and β6 proteasome subunits are clearly characterized, as revealed by SEM analysis, by gene-specific abnormal eye phenotypes, with fused facets and disturbed bristle arrangements representing the most prominent features (Figs. 1Be–l and 2), compared to GAL4-ninaE.GMR control flies (Figs. 1Ba–d). Specifically, flies with reduced expression of Rpn1 and Rpn2 19S cap proteasome subunits present under Stereo-microscopic view with eyes lacking large areas of orange pigment cells (Figs. 1Be and i) and under SEM examination with rough eye phenotypes, hyperplastic areas and loss of inter-ommatidial bristles (Figs. 1Bf, g′, j and k). In addition, the lenses of numerous individual ommatidia are entirely missing (Figs. 1Bg and k). Semi-thin sections reveal ommatidia with severely altered cell morphologies, mainly characterized by loss of pigment cells and extensive degeneration of photoreceptor clusters (Figs. 1Bh and l), especially in the Rpn1 mutant line. Intriguingly, the eye-specific overexpression of Rpn6 RNAi causes embryonic lethality and therefore no adult flies can be born. Downregulation of the α5 proteasome subunit of 20S catalytic core results in eyes exhibiting areas of black pigmentation (Fig. 2a), rough eye phenotypes, reduced number of bristles, ommatidia without lenses, disruption of eye structural organization (Figs. 2b and c) and ommatidia with reduced number of photoreceptors (Fig. 2d). The eyes developed after β5 or β6 core proteasome subunit downregulation proved to carry necrotic foci (Fig. 2e) and loss of orange pigment (Fig. 2i), respectively. They are characterized by mild rough appearance with collapsed and fused ommatidia, loss of inter-ommatidial bristles and disturbance of architectural integrity (Figs. 2f, g, j and k). After semi-thin sections, ommatidia present with reduced differentiation levels and loss of pigment cells, while the number of underlying photoreceptor cells is strongly diminished, so that many ommatidia lose their typical structure, and appear with severely misplaced rhabdomeres (Fig. 2h) and empty areas likely reflecting cellular detachment events (Fig. 2l), respectively.

**Figure 3:**
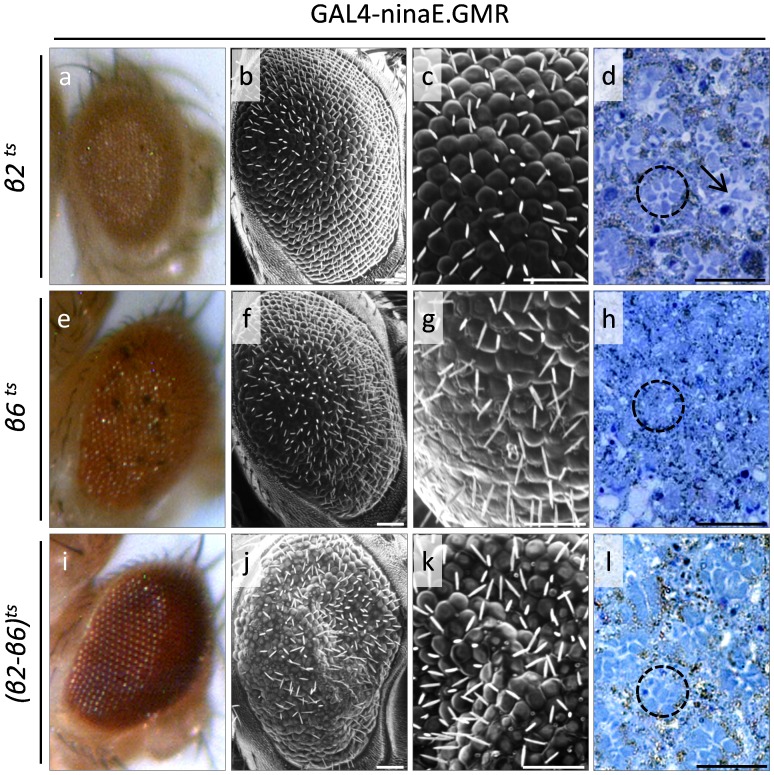
The β2 and β6 20S proteasome core subunits play essential roles in fly eye development. Stereo-microscopical (a, e and i), Optical (d, h and l {semi-thin sections}) and Scanning Electron Microscopy (SEM) (b, c, f, g, j and k) images, demonstrating the critical contribution of β2 and β6 20S proteasome core subunits in fly eye morphogenesis. (a–d) GAL4-ninaE.GMR/UAS-Prosbeta21 double transgenic fly eyes, overexpressing the conditionally (temperature sensitive {ts}) mutant β2^ts^ proteasome core protein form. (e–h) GAL4-ninaE.GMR/UAS-Pros261 double transgenic fly eyes, characterized by upregulated mutant β6^ts^ expression levels. (i–l) GAL4-ninaE.GMR/UAS-Prosbeta21 - UAS-Pros261 triple transgenic flies, bearing increased eye-specific contents of mutant (defective) β2^ts^ and β6^ts^ proteasome subunit forms. Dashed circle: ommatidium. Arrow: lesion area(s). Scale Bars: 50 µm.

Temperature sensitive (ts) transgenic flies (raised at 29°C, where β2^ts^ and β6^ts^ proteins can obtain their respective mutant characters), carrying non-functional either β2^ts^ or β6^ts^, or both β2^ts^ and β6^ts^ subunits of the 20S proteasome particle, appear to also produce dysmorphic eyes, as revealed through SEM and semi-thin section protocols (Fig. 3). Eye-specific overexpression of a defective β2^ts^ core proteasome subunit leads to ommatidia that lack orange pigmented granules (Fig. 3a), rough eye phenotypes, ommatidia without lenses, partial loss of inter-ommatidial bristles and disrupted ommatidial array (Figs. 3b and c). Semi-thin sections disclosed more severe phenotypes, with the almost complete photoreceptor and ommatidia degeneration being one of the predominant defects (Fig. 3d). Flies bearing a non-functional β6^ts^ proteasome subunit present with mild rough eye phenotype, due to a disrupted ommatidial array structure and partial loss or disorganization of inter-ommatidial bristles (Figs. 3e–g). Semi-thin sections reveal areas of ommatidia with differentiation and morphology defects, and ommatidia with reduced number of photoreceptors (Fig. 3h). Eyes produced by flies carrying both β2^ts^ and β6^ts^ defective proteasome subunits demonstrate a likely additive and, thus, more severe mutant phenotype with areas of black pigmentation (Fig. 3i), collapsed and fused ommatidia, disturbance of structural integrity and partial loss of inter-ommatidial bristles (Figs. 3j and k). Semi-thin sections revealed serious ommatidia degeneration and major reduction in the number of photoreceptor neurons (Fig. 3l).

**Figure 4:**
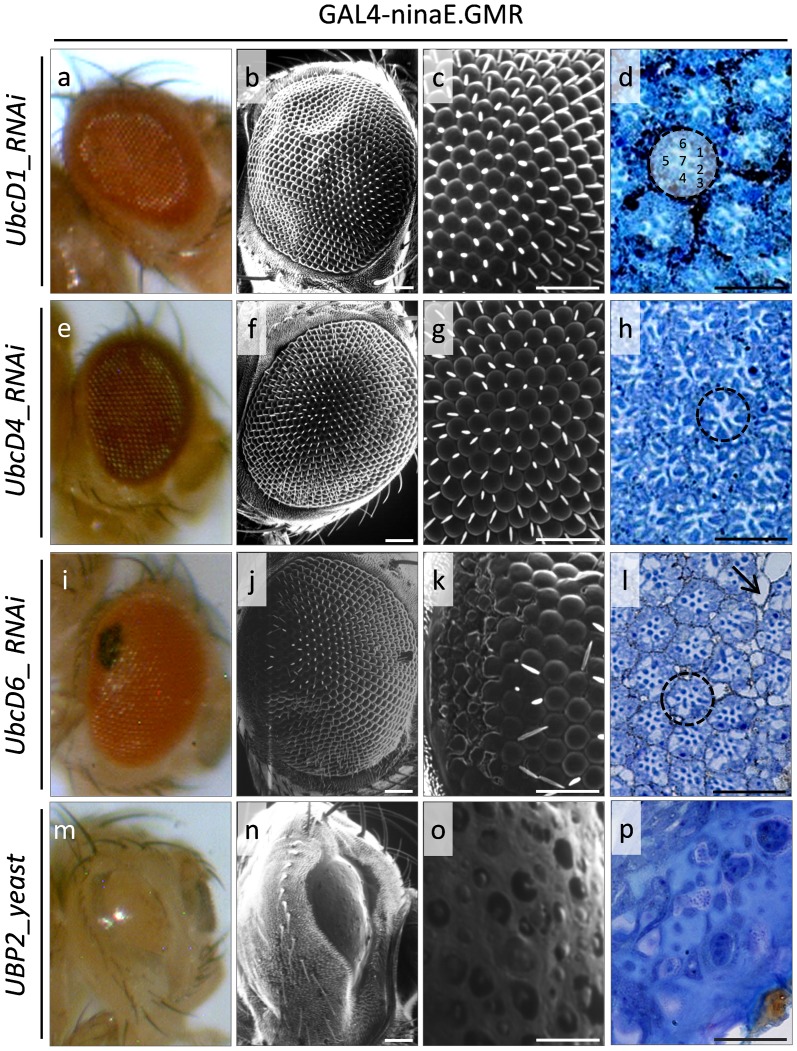
Ubiquitin homeostasis controls Drosophila eye structure in a gene-dependent manner. Stereo-microscopical (a, e, i and m), Optical (d, h, l and p {semi-thin sections}) and Scanning Electron Microscopy (SEM) (b, c, f, g, j, k, n and o) images, illustrating the morphology and architectural pattern of fly eye in cellular environments containing reduced protein contents of UbcD1, UbcD4 and UbcD6 E2 ubiquitin conjugating enzymes (a–l), or overexpressed UBP2 (-yeast- ubiquitin protease) protein levels (m–p) (arrow: lesion area{s}). (a–d) GAL4-ninaE.GMR/UAS-UbcD1_RNAi double transgenic fly eyes, likely carrying reduced UbcD1 protein contents. (e–h) GAL4-ninaE.GMR/UAS-UbcD4_RNAi double transgenic fly eyes, characterized by downregulated UbcD4 enzyme expression levels. (i–l) GAL4-ninaE.GMR/UAS-UbcD6_RNAi double transgenic flies, containing diminished UbcD6 cellular protein amount specifically in the eye. (m–p) GAL4-ninaE.GMR/UAS-UBP2.D double transgenic fly eyes, overexpressing the UBP2 (yeast) ubiquitin protease (note the highly dysmorphic and eyeless-like phenotype {n and o}). Dashed circle: ommatidium. 1–7: photoreceptor cells. Scale Bars: 50 µm.

Double transgenic flies with reduced expression levels of the UbcD1, UbcD4 and UbcD6 proteasome critical regulators seem to generally retain physiological structural and organizational patterns of eye development, as examined via SEM analysis, although mild deformities can be recognized by semi-thin section examination. Specifically, RNAi-mediated downregulation of UbcD1 and UbcD4 E2 ubiquitin conjugating enzymes is directly associated with eyes of unharmed ommatidia organization (Figs. 4a–c and e-g). However, semi-thin section analysis reveals ommatidia characterized by altered cell shapes, reduced differentiation levels and partial loss of pigment cells, all major features that significantly contribute to an unusual ommatidium structure (Figs. 4d and h). Interestingly, the external eye morphology of flies carrying reduced UbcD6 enzyme levels exhibits (comparatively) a more severely defective profile of eye architecture, with a predominant area of black pigmentation (Fig. 4i). This region, after SEM examination, appears with collapsed or fused ommatidia and partial loss of inter-ommatidial bristles (Fig. 4j and k). Staining of photoreceptor neurons demonstrates a rather wild-type ommatidia structure, although empty areas likely reflecting cellular detachment and/or vacuolar-type degeneration can be also observed (Fig. 4l).

GAL4-ninaE.GMR-mediated overexpression of UBP2 -yeast- ubiquitin protease is tightly associated with a dramatic elimination of all ommatidia vision-units, in the double transgenic adult flies, producing smooth and eyeless-like phenotypes (Figs. 4m–p). Remarkably, mutant flies present with very small, narrow and smooth eyes characterized by complete absence of developed ommatidia. Semi-thin sections disclose total loss of photoreceptor neurons, which critically contributes to the generation of highly dysmorphic eyes (Fig. 4p).

Conclusively, RNAi-mediated disruption of fly ubiquitin/proteasome machinery induces defects in eye morphology and architectural organization that follow gene-specific dysmorphic profiles. Typical deformities include, among others, loss of pigment cells, rough eye phenotypes, disruption of ommatidial arrays, collapsed or fused ommatidia, ommatidia without lenses, degenerated photoreceptors with small or missing rhabdomeres, loss of photoreceptor neurons, vacuolar-type degeneration and in the most devastating case (UBP2 downregulation) complete absence of ommatidia.

### Ubiquitin/proteasome Downregulation Induces Severe Morphogenetic Defects in Fly wing: a Gene-specific Process

To investigate the functional importance of ubiquitin/proteasome machinery in a distinct from the eye developmental system, we overexpressed defective, or heterologous, critical proteasome determinants or selected RNAi species, using the strongly and preferentially expressed in the wing disc P{GawB}BxMS^1096^ GAL4 genetic driver (Fig. 1Ab), and subsequently examined fly wings for visible dysmorphic structures. The obtained phenotypes ranged from wings with physiological morphology to wings that were severely wrinkled and architecturally collapsed.

**Figure 5:**
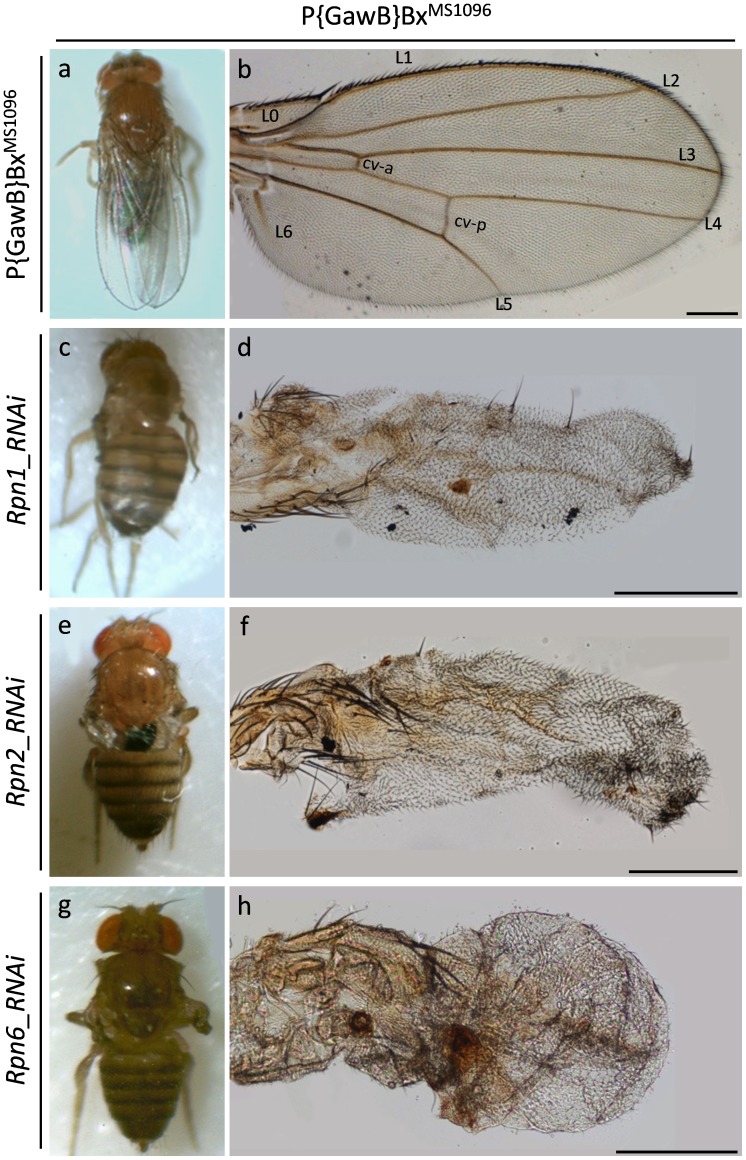
RNAi-mediated disruption of the 19S proteasome regulatory cap results in highly dysmorphic fly wings. Stereo-microscopical (a, c, e and g) and Optical (b, d, f and h) images, demonstrating severely dysmorphic wing phenotypes obtained under Rpn1, 2 and 6 deficient cellular conditions. (a and b) P{GawB}Bx^MS1096^ single transgenic adult fly wings (utilized as control organ-tissues), structured by a marginal vein encompassing the length of the anterior wing margin, four longitudinal veins (L2–L5), two incomplete longitudinal veins (L1 and L6) and two transverse veins (cv-a {anterior cross-vein} and cv-p {posterior cross-vein}). (c and d) P{GawB}Bx^MS1096^/UAS-Rpn1_RNAi double transgenic fly wings, characterized by reduced expression of Rpn1 19S proteasome subunit. (e and f) P{GawB}Bx^MS1096^/UAS-Rpn2_RNAi double transgenic fly wings, carrying downregulated Rpn2 protein levels. (g and h) P{GawB}Bx^MS1096^/UAS-Rpn6_RNAi double transgenic flies, producing wings with decreased Rpn6 cellular contents. Scale Bars: 200 µm.

Insect wings are composed of only 2 cell layers, one on the dorsal and the other on the ventral wing surface. Wing veins comprise the most characteristic structures of the wing and are formed within the one or the other cell layer, though they can pass through from one wing surface to the other [3], [17]. Veins confer structural rigidity to the wing and also enclose conducts in which the haemolymph can circulate, while they may carry trachea and axons [18]. They appear as longitudinal stripes of cells that differentiate to darkly pigmented cuticle and are more packed than intervein cells. In *Drosophila*, there are four longitudinal veins (L2–L5) that typically run from the base to the apex of the wing and are distributed in species-specific two-dimensional patterns [4], [19], and two transverse veins (anterior and posterior cross-veins) that connect the longitudinal L3–L4 and L4–L5 ones, respectively (Fig. 5b). In addition, the *Drosophila* wing contains a marginal vein encompassing the length of the anterior wing margin and two incomplete longitudinal veins, one in the anterior (L1) and one in the posterior (L6) compartment. In the holometabolous insects, longitudinal veins form first, while cross-veins that connect the longitudinal ones develop somewhat later [18], [20], [21]. The ordered contribution of cell signalling and transcriptional regulation determine how and where vein cells appear and differentiate [22]. Therefore, wing-vein development is an excellent model system for illuminating epithelial-morphogenesis pathways and characterizing novel genes that are critically implicated in epithelial cell proliferation and differentiation.

RNAi-mediated downregulation of Rpn1, Rpn2 and Rpn6 subunits of the 19S proteasome regulatory cap is tightly associated with highly damaged wings, therefore documenting the essential role of 19S regulatory cap components in wing morphogenesis. Specifically, the mutant wings are small in size and are characterized by severely wrinkled structural patterns accompanied by failure of vein cell differentiation (Figs. 5c–h). In addition, they are mildly spoon-shaped, compared to *Drosophila* control fly wings (Figs. 5a and b), suggesting the implication of Rpn-dependent cell proliferation or viability defects.

**Figure 6:**
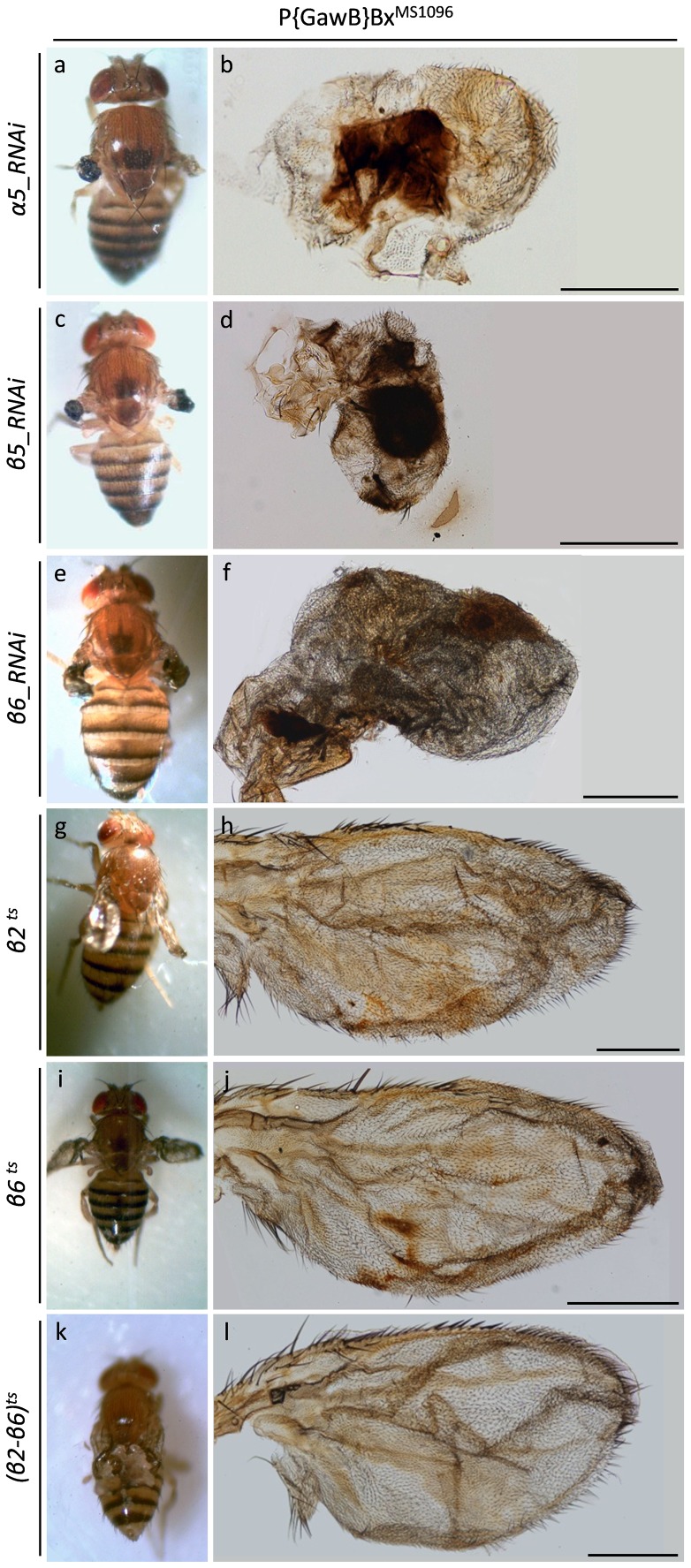
Disintegration of 20S proteasome core particle leads to fly wing dysmoprhia. Stereo-microscopical (a, c, e, g, i and k) and Optical (b, d, f, h, j and l) images, illustrating the critical roles of α- and β-based rings of the 20S proteasome catalytic core in wing morphogenesis. (a and b) P{GawB}Bx^MS1096^/UAS-alpha5_RNAi double transgenic fly wings, carrying reduced cellular contents of α5 proteasome subunit. (c and d) P{GawB}Bx^MS1096^/UAS-dbeta5_RNAi double transgenic fly wings, characterized by downregulated β5 expression levels. (e and f) P{GawB}Bx^MS1096^/UAS-beta6_RNAi double transgenic flies, producing wings with diminished amount of β6 20S proteasome protein. (g and h) P{GawB}Bx^MS1096^/UAS-Prosbeta21 double transgenic fly wings, overexpressing the conditionally (temperature sensitive {ts}) mutant β2^ts^ proteasome subunit. (i and j) P{GawB}Bx^MS1096^/UAS-Pros261 double transgenic fly wings, carrying increased levels of conditionally mutant β6^ts^ protein specifically in the wing. (k and l) P{GawB}Bx^MS1096^/UAS-Prosbeta21 - UAS-Pros261 triple transgenic flies, bearing dysmorphic wings due to organ-specific overexpression of defective (conditionally mutant) β2^ts^ and β6^ts^ 20S proteasome components. Scale Bars: 200 µm.

Reduced expression of α5, β5 and β6 20S core proteasome subunits induces formation of folded, self-looped and blistered wings, also featured by loss of wing veins and adjacent tissues (Figs. 6a–f). In accordance, overexpression of conditionally (temperature sensitive) defective either β2^ts^ or β6^ts^, or both β2^ts^ and β6^ts^ proteasome subunits strongly impairs wing development and the produced wrinkled wings fail to obtain physiological wing vein differentiation (Figs. 6g–l).

**Figure 7:**
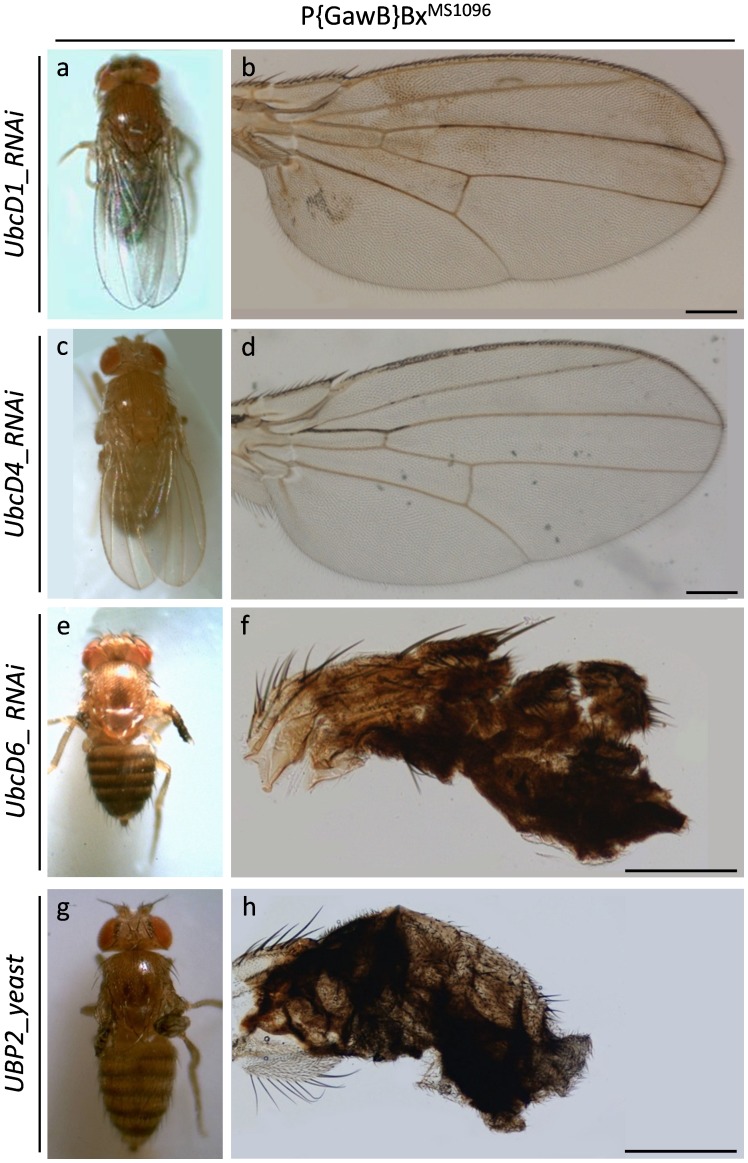
Malfunction of ubiquitin trafficking induces gene-specific defects in Drosophila wing development. Stereo-microscopical (a, c, e and g) and Optical (b, d, f and h) images, revealing the gene-specific contribution of UbcD1, 4, 6, and UBP2 ubiquitin homeostasis regulators in fly wing morphogenesis. (a and b) P{GawB}Bx^MS1096^/UAS-UbcD1_RNAi double transgenic fly wings, characterized by likely reduced UbcD1 protein contents. (c and d) P{GawB}Bx^MS1096^/UAS-UbcD4_RNAi double transgenic fly wings, carrying likely downregulated UbcD4 expression levels. (e and f) P{GawB}Bx^MS1096^/UAS-UbcD6_RNAi double transgenic flies, producing highly dysmorphic wings due to organ-specific and diminished cellular amount of UbcD6 E2 ubiquitin conjugating enzyme. (g and h) P{GawB}Bx^MS1096^/UAS-UBP2.D double transgenic flies, overexpressing the UBP2 -yeast- ubiquitin protease specifically in the wing. Arrow: lesion area(s). Scale Bars: 200 µm.

Double transgenic flies, carrying downregulated expression levels of UbcD1 and UbcD4 E2 ubiquitin conjugating enzymes, prove to retain undisturbed patterns of wing morphogenesis, without any detectable structural deformity (Figs. 7a–d). However, functional loss of UbcD6 family member severely disrupts wing development and the -small- ruined wings are basically characterized by total lack of margin and L1–L6 vein differentiation, and also by absence of mechanosensory and chemosensory bristles (Figs. 7e and f).

Finally, wing disc-targeted overexpression of the UBP2 -yeast- ubiquitin protease results in small rudimentary wings, without distinguishable vein differentiation (Figs. 7g and h).

Conclusively, wing disc-specific functional disruption of ubiquitin/proteasome machinery by RNAi-mediated elimination of (a) Rpn1, Rpn2 and Rpn6 19S cap proteasome subunits, (b) α5, β5 and β6 20S core proteasome subunits and (c) UbcD6 proteasome critical regulator, together with overexpression of (d) -conditionally- defective either β2^ts^ or β6^ts^, or both β2^ts^ and β6^ts^ 20S core proteasome subunits and (e) UBP2 -yeast- ubiquitin protease, are strongly associated with fly wing dysmorphia, resulting in severely folded and blistered wings with loss of vein differentiation or in wings that are totally atrophic, therefore disclosing the essential and component-dependent morphogenetic role of ubiquitin/proteasome activity in physiological wing development.

### Autophagy Suppression Results in Mild Morphogenetic Defects during wing, but Not Eye, Development

**Figure 8:**
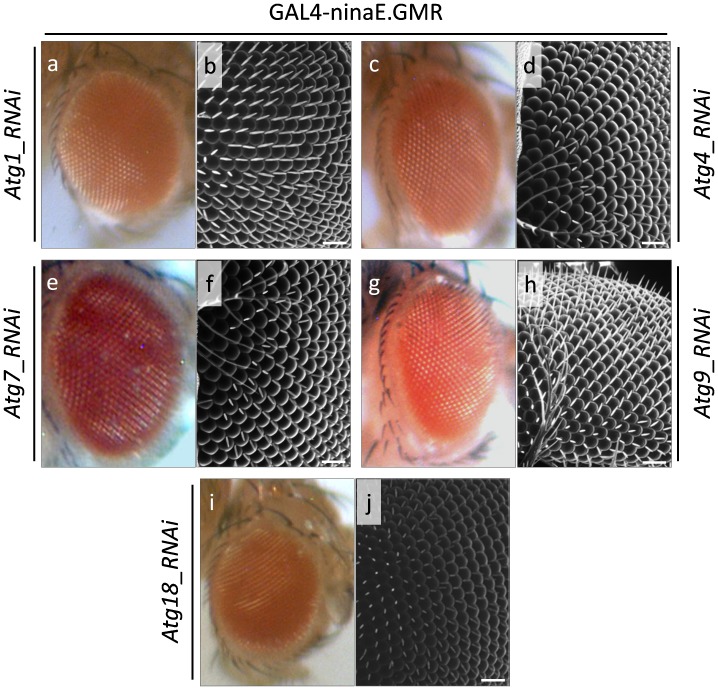
RNAi-mediated elimination of critical autophagic proteins does not harm fly eye architectural structure. Stereo-microscopical (a, c, e, g and i) and Scanning Electron Microscopy (SEM) (b, d, f, h and j) images, illustrating the surface morphology and structural organization of Drosophila compound eye in Atg1, 4, 7, 9 and 18 deficient cellular environments. (a and b) GAL4-ninaE.GMR/UAS-Atg1_RNAi double transgenic fly eyes, characterized by likely reduced Atg1 protein contents. (c and d) GAL4-ninaE.GMR/UAS-Atg4_RNAi double transgenic fly eyes, carrying downregulated Atg4 expression levels. (e and f) GAL4-ninaE.GMR/UAS-Atg7_RNAi double transgenic fly eyes, likely producing diminished amount of Atg7 autophagic protein. (g and h) GAL4-ninaE.GMR/UAS-Atg9_RNAi double transgenic fly eyes, probably containing decreased Atg9 protein levels. (i and j) GAL4-ninaE.GMR/UAS-Atg18_RNAi double transgenic fly eyes, likely developed in an Atg18 deficient cellular background. Scale Bars: 50 µm.

Unlike disruption of proteasome activity, the RNAi-mediated elimination of Atg1, Atg4, Atg7, Atg9 and Atg18 critical autophagic proteins reveals their non-essential, or redundant, functional roles in *Drosophila* eye morphogenesis (Fig. 8). Eyes with reduced Atg (1, 4, 7, 9 and 18) protein levels present with wild-type morphology and undisturbed ommatidium array, as documented by optical and scanning electron microscopy approaches (Figs. 8Aa–j).

**Figure 9:**
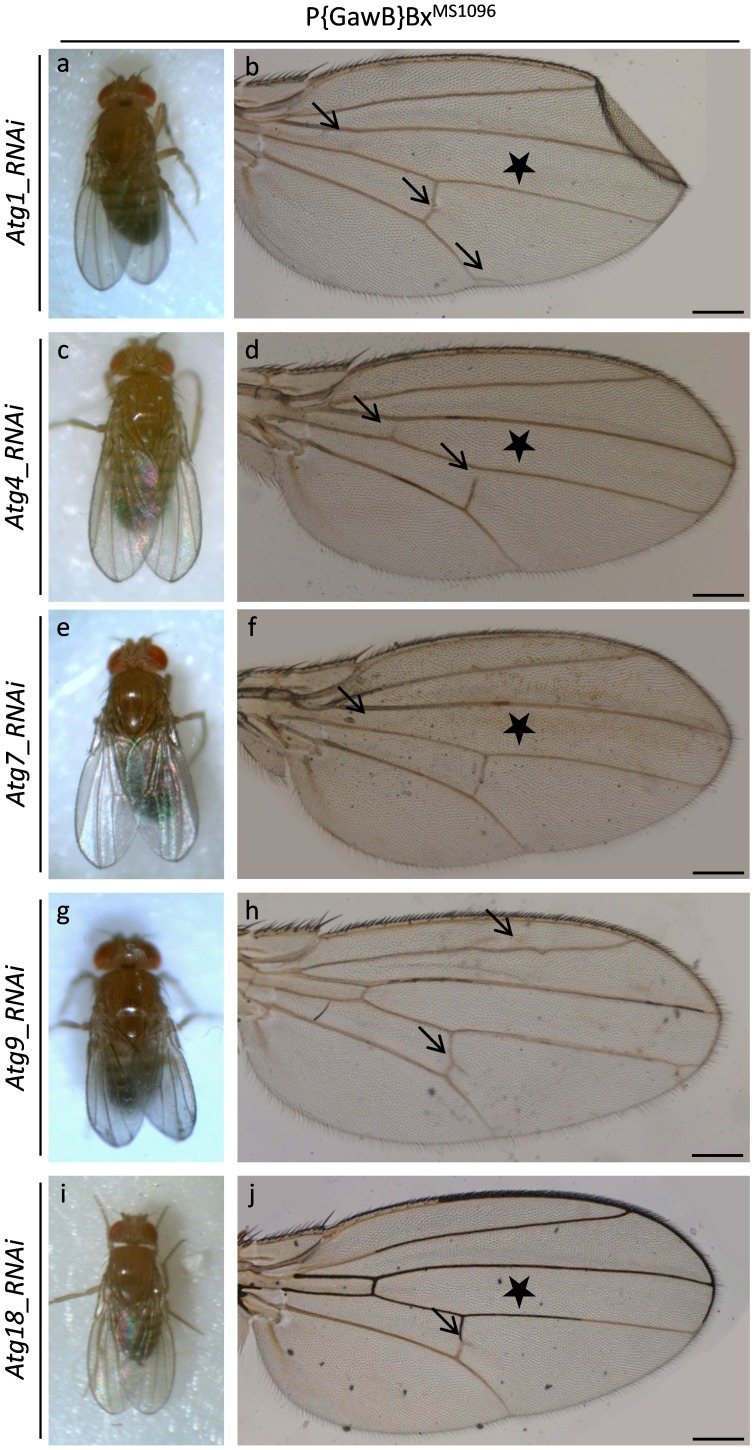
Downregulation of autophagy is associated with mild Drosophila wing lesions. Stereo-microscopical (a, c, e, g and i) and Optical (b, d, f, h and j) images, demonstrating that, attenuation of autophagy can induce only marginally pathological phenotypes of fly wing formation (a–j) (arrows). (a and b) P{GawB}Bx^MS1096^/UAS-Atg1_RNAi double transgenic fly wings, characterized by reduced Atg1 protein contents. (c and d) P{GawB}Bx^MS1096^/UAS-Atg4_RNAi double transgenic fly wings, produced in Atg4 deficient cellular environments. (e and f) P{GawB}Bx^MS1096^/UAS-Atg7_RNAi double transgenic fly wings, carrying downregulated Atg7 expression levels. (g and h) P{GawB}Bx^MS1096^/UAS-Atg9_RNAi double transgenic fly wings, developed under cellular conditions of attenuated Atg9 autophagic protein synthesis. (i and j) P{GawB}Bx^MS1096^/UAS-Atg18_RNAi double transgenic fly wings, bearing decreased amount of Atg18 protein. Asterisks (b {Atg1}, d {Atg4}, f {Atg7} and j {Atg18}): double (heterozygote) transgenic fly populations, containing both wild-type and mutant (at a low frequency) wing phenotypes. Scale Bars: 200 µm.

**Figure 10:**
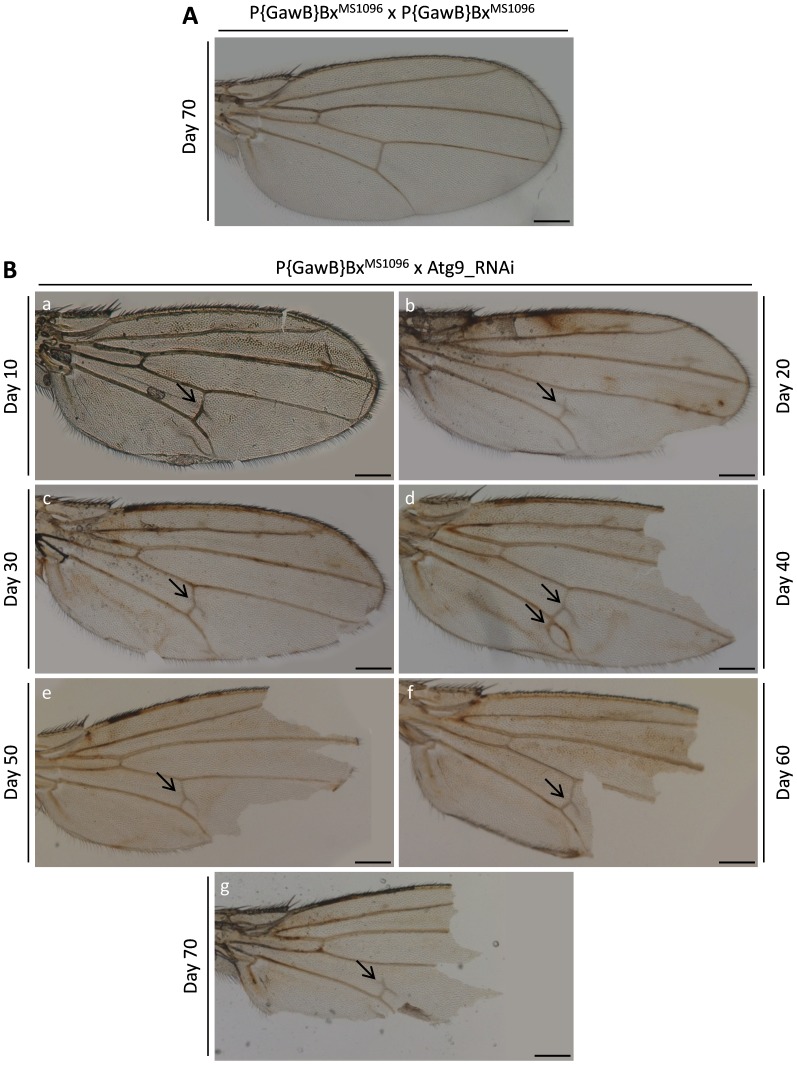
RNAi-mediated reduction of Atg9 protein levels is tightly associated with loss of wing’s structural integrity in aged Drosophila flies. (A) Wings from 70 days-old single (homozygote) transgenic (both male and female) flies carrying only the P{GawB}Bx^MS1096^ genetic driver present (in their vast majority) with a generally physiological morphology and structure. (B) Optical images (a–g) of double (heterozygote) transgenic wings, carefully isolated from 10 to 70 days-old female flies that were produced by Atg9_RNAi homozygote females crossed with P{GawB}Bx^MS1096^ homozygote males, clearly demonstrate the age-dependent structural fragility of the organ (broken wings). Arrows: wing dysmorphic features. Scale Bars: 200 µm.

In small contrast to eye morphogenesis, RNAi-induced downregulation of critical proteins regulating autophagy (Atg1, 4, 7, 9 and 18) proves to harm fly wing development and architecture in an apparently mild way. Even though the produced mutant wings appear generally normal in size and shape, incidents characterized by loss of vein tissue and/or ectopic vein patches can be observed (Fig. 9). Specifically, reduced expression of Atg1 protein results in mixed fly wing phenotypes, with flies presented (at a low frequency) with abnormal wings that lack the anterior cross-vein and carry ectopic vein tissue in the posterior cross-vein and distal tip of L5 vein (Figs. 9a and b). Functional elimination of Atg4 protein also causes mixed wing phenotypes to flies, with either physiological structure or vein structural gap(s) in the anterior and posterior cross-veins (at a low frequency) (Figs. 9c and d). Similarly, downregulation of Atg7 autophagic protein produces, besides wings with normal architecture, wings with loss of the anterior cross-vein (at a low frequency) (Figs. 9e and f). Wing-specific reduction of Atg9 protein expression levels is rather always associated with ectopic vein patches formation in the posterior cross-vein, loss of anterior cross-vein (in some cases {i.e. Fig. 10Bb}), along with occasionally disturbed development of the L2 and L3 longitudinal veins (Figs. 9g and h, Fig. 10B and data not shown). Finally, downregulation of Atg18 autophagic protein leads (at a low frequency) to extra vein tissue growth in the posterior cross-vein of fly wing (Figs. 9i and j).

### Atg9 Autophagic Protein Controls Fly wing Fragility in an Age-dependent Manner

The herein presented data indicate a rather non-essential (or redundant) role of autophagy during wing development in young adult (2 or 3 days old) flies. Since previous reports have clearly underscored the beneficial role of autophagy during stress and aging (for a detailed review see [23]), we next examined wing structure and morphology in flies carrying reduced Atg (1, 4, 7 and 9) protein levels every 10 days of lifetime for an experimental period of 10 to 70 days. Among the Atg double (heterozygote) transgenic lines tested, only Atg9_RNAi mutant flies proved to obtain a pathogenic wing phenotype (compared to the normal {control} one), mainly characterized by an age-dependent strong fragility of the organ. The RNAi-mediated downregulation of Atg9 autophagic protein is tightly associated with loss of wing’s structural integrity in aged flies, with their entire population carrying severely broken wings after the 50^th^ day of lifetime (Figs. 10Ba–g). In contrast, 70 days-old single (homozygote) transgenic flies bearing only the respective P{GawB}Bx^MS1096^ genetic driver present (in their vast majority) with generally undisturbed wing morphology and structure (Fig. 10A).

## Discussion

The visual system of *Drosophila melanogaster* is a powerful genetic model for dissecting the molecular mechanisms underlying neurodegenerative diseases [24], [25]. On the other hand, *Drosophila* wing venation is a classical developmental model for defining genetic interactions involved in cell proliferation, cell-cell communication, and cell differentiation [4], [26]. In this context, and in an effort to investigate the *in vivo* developmental dynamics of the two major proteolytic mechanisms, proteasome and autophagy, in a cell, we examined the morphogenetic role of critical proteasome subunits, together with selected proteasome regulators and autophagy components, in the process of *Drosophila* compound eye and wing formation.

It has been reported that the GMR-GAL4 genetic driver, besides fly eye discs, can also operate in other tissues (i.e. brain, trachea and leg discs) and thus caution should be taken in the interpretation of certain pathogenic phenotypes, such as lethality and behavioral defects [27]. In contrast, for eye development studies, it is widely used and generally accepted as an eye-specific driver able to overexpress or downregulate (through RNAi) native proteins of our preference [13], [28], [29]. Similarly, the P{GawB}Bx^MS1096^ driver examined here represents a valuable genetic tool for reliably dissecting the mechanisms of wing morphogenesis, as it has been previously shown by a number of different groups [30]–[32]. To this direction, each driver’s (GAL4-ninaE.GMR or P{GawB}Bx^MS1096^) organ specificity and absence of toxicity are herein documented by the (a) “green-fluorescent” and physiological phenotypes obtained from the -control- UAS-2xEGFP crosses (Figs. 1Aa and b, and data not shown), (b) undisturbed (eye or wing) structures generated by homozygote single transgenic lines carrying only the respective driver (Figs. 1Ba–d, 5a and b, and 10A) and (c) production of certain heterozygote double transgenic flies characterized by non-pathogenic organ phenotypes (Figs. 4a–h, 7a–d and 8a–j).

Ommatidial development begins during the third larval stage within the eye imaginal disc and retinal differentiation is initiated by hedgehog (hh) signaling, which “cross-talks” to decapentaplegic (dpp) and wingless (wg) signaling pathways [33], [34]. In our study, the majority of transgenic flies with downregulated proteasome determinant expression levels presented with either a rough eye phenotype or severely dysmorphic eye structure, mainly carrying a disrupted ommatidial array, and following a subunit- and regulator-specific pattern of dysmorphia. Ommatidia appeared collapsed, fused or without lenses, with degenerated photoreceptors and small or missing rhabdomeres. More specifically, given that both Rpn1 and Rpn2 19S proteasome subunit reduced contents are able to induce distinct dysmorphic eye phenotypes, they might probably recognize different groups of ubiquitinated proteins in a rather essential, and non-redundant, manner. Similarly, loss of the α5 20S proteasome core component cannot be compensated by the β-type subunits and *vice versa*. Moreover, each β ring protein examined (2, 5 and 6) proved to carry indispensable functions for fly eye development, even though β2 and β6 could share overlapping modes of action, since the dysmorphic structure obtained by overexpressing both β2^ts^ and β6^ts^ mutant (defective) subunits presented with comparably more dramatic features than the β2^ts^ or β6^ts^ single respective one. To the same direction, in contrast to UbcD6 that seemed to significantly contribute to the process of fly eye development, a redundant role could be assigned to UbcD1 and UbcD4 proteasomal regulators. Alternatively, UbcD6 (but not UbcD1 and 4) might selectively conjugate ubiquitin moieties to certain eye-specific protein substrates, whose targeting to proteasome for degradation is absolutely critical for normal development. The ability of β5 (and β6) elimination to cause stronger defects, compared to the α5 downregulation-induced respective ones, in eye cellular architecture dictates the subunit-dependent ability of proteasome to counterbalance the loss of one (or more) of its structural components.

The physiological importance of proteasome integrity during *Drosophila* eye development is further supported by a previous study in which depletion of Pros26.4 (encodes the Rpn2 orthologue in *Drosophila*) within the differentiating eye field resulted in fused ommatidia and a graded loss of internal retinal tissue, while a strong increase in cell death was also observed in the third instar larval eye discs [35]. E3 ubiquitin ligases have been also shown to control both the differentiating signal and the proliferation regulation of *Drosophila* eye development, through distinct F-box proteins (Slimb and Ago, respectively) [36].

Ubiquitin-specific proteases (UBPs) are classified among the most important determinants in eye morphogenesis. Mutations in several UBP family members in humans have been associated with neurodegeneration, cancer and inflammatory diseases [37]–[39], suggesting that UBP activities must be tightly controlled in order to maintain the necessary levels of ubiquitination in the cell. In our system, overexpression of the yeast UBP2 ubiquitin protease proved to cause a dramatic elimination of all ommatidia structures in the adult fly eye, producing an eyeless-like phenotype. Despite its fungal evolutionary origin, it seems that UBP2 can efficiently recognize and cleave the respective cellular substrates in the fly eye heterologous environment, therefore revealing the conserved and tightly regulated functional nature of ubiquitin trafficking in likely all eukaryotic kingdoms.

In *Drosophila*, the most extensively studied UBP family member is encoded by the *fat facets* (*faf*) gene and is specifically required for eye development [40]. *Drosophila faf* null mutants are characterized by abnormal eye morphology, with the most prominent defect being the formation of more than eight photoreceptors in each facet [41]. The role of faf protein has been also implicated in Notch signaling, where it enhances Delta endocytosis in order to promote Notch signaling and ensure the correct recruitment of photoreceptor precursors [42]. In this context, faf de-ubiquitinates and stabilizes Liquid facets (Lqf) protein (homologue of vertebrate epsin), which in turn mediates the endocytosis of Delta receptor, a critical process for cell patterning [42]–[44]. Therefore, overexpression of yeast UBP2 in *Drosophila* eye could -among others- reduce the ubiquitinated Lqf cellular load, subsequently inducing Delta receptor endocytosis and Notch signaling hyper-activation, which might be able to suppress photoreceptor neuron differentiation, thus resulting in the eyeless-like phenotype observed.

Neuralized (Neur), an E3 ubiquitin ligase, is also required for Delta internalization in wing and eye discs [45], [46]. *Neur* adult eye mutant phenotype resembles the one of *faf* and *Lqf^FDD9^* mutants, therefore indicating that Neur and Lqf might work together to stimulate Delta endocytosis and that all three (Neur, Lqf and faf) proteins function in the same direction in this pathway [42]. It is suggested that Lqf facilitates Delta endocytosis by binding to Delta after its ubiquitination by Neur [42]. Hence, UBP2 could stabilize Lqf, which in turn recognizes the Neur-induced ubiquitinated form of Delta receptor, therefore promoting its (Delta) internalization in a fly photoreceptor cellular environment. UBP64, another de-ubiquitinating enzyme in *Drosophila*, controls cell fate in developing eye through stabilization of the transcriptional repressor tramtrack (TTK). Ubp64 hypomorphic mutants displayed a rough eye phenotype, with the defective eyes being characterized by disorganized facets and bristles [47]. Overexpression in eye imaginal discs of the dUCH de-ubiquitinating enzyme induced a rough eye phenotype in the adult fly, specifically impairing R7 photoreceptor cell differentiation, by downregulating the MAPK signaling pathway [48]. To this direction, UBP2 overexpression could result -among others- in MAPK signaling repression, which might subsequently inhibit R7 development, thus critically contributing to the severe fly eye dysmorphia observed. It seems that the balance between ubiquitination and de-ubiquitination of several cellular proteins essentially controls *Drosophila* eye morphogenesis, while its deregulation results in highly dysmorphic ommatidium structures.

Similarly to *Drosophila* eye morphogenesis, the main drivers of wing development are the hedgehog (hh), Notch, wingless (wg) and decapentaplegic (dpp) signaling pathways [4]. Activation of hh- and wg-dependent signaling leads to increased stability of the transcription factors Cubitus interruptus (Ci) and Armadillo (Arm), respectively. Both hh and wg signaling functions are negatively regulated by the *slimb* gene, which encodes a conserved F-box/WD40-repeat protein that acts by targeting Ci and Arm transcription factors for ubiquitination and proteolysis in the proteasome [49]. Ci protein stability, and therefore hh signaling potency, can be also downregulated by the *dUba3* and *dUbc12* genes, each belonging to functionally distinct (E1 and E2) UPS (ubiquitin/proteasome system) component group [50]. Reduced expression of dUba3 enzyme by RNAi-based technology in the wing discs at restrictive temperature resulted in lethality at the pupal stage, while at permissive temperature some flies survived into the adulthood but were characterized by folded and damaged wing blades [50]. It has been also reported that Ci stability can be regulated by a number of distinct E3 ligases able to target the protein to proteasome [51]. Similarly, UbcD6 E2 enzyme could promote Ci ubiquitination, while its (UbcD6) RNAi-mediated elimination -among others- could induce Ci protein stabilization and hh signaling hyper-activation, likely causing the severe fly wing dysmorphia observed. The putative ability of UbcD1 and UbcD4 family members to ubiquitinate substrates of similar cellular functions, or even the very same substrates, might be tightly associated with the normal phenotypes obtained upon their (UbcD1 and 4) wing-specific downregulation. However, an alternative scenario of tissue-dependent transcriptional repression of *UbcD1* and *UbcD4* genes specifically in the fly wing cannot be excluded.

Regarding dpp signals, they emanate from activated cell surface receptors and are transduced by intracellular Smad proteins, with MAD being the founding member of the family [52]. The dpp signaling pathway can be specifically inhibited by dSmurf protein, a *Drosophila* E3 ligase that targets phosphorylated MAD transcription factor to proteasome-mediated degradation. Targeted overexpression of dSmurf in wing disc causes disruption of dpp-dependent patterning of the organ, while pharate adult flies removed from the pupal case show rudimentary wings [52].

Therefore, any structural disintegration of either 19S cap (through Rpn1, Rpn2 or Rpn6 downregulation) or 20S core (through α5, β2, β5 or β6 downregulation) fly proteasome particle likely increases ubiquitinated Ci, Arm and MAD protein cellular loads, and deregulates signaling activities of the respective pathways, ultimately resulting in the development of dysmorphic wing structures. Given that the RNAi-mediated functional knockout of α5, β5 or β6 proteasome subunits is associated with more dramatically malformed wing phenotypes compared to the ones evolved by β2^ts^, β6^ts^ or β2^ts^ and β6^ts^ overexpression, it proves that, during wing morphogenesis, proteasome can more efficiently tolerate a mutant and defective component than a loss of subunit committed to assemble the 20S core particle.

Zhang *et al.*, by employing the C96-GAL4 driver, which confers a relatively lower but restricted expression of RNAi transgenes along the presumptive wing margin, identified four potential de-ubiquitinase (DUB) regulators of Notch signaling. The knockdown of each one of them through RNAi technology resulted in significantly altered adult wing margin formation and/or excessive bristle development, likely attributed to the reduced activity of Notch pathway mediators, therefore suggesting a positive role of DUB enzymes in Notch signaling and *Drosophila* wing patterning [53]. To this direction, the UBP2 protein overexpression in fly wing -among others- could severely derail Notch signaling function (hyper-activation), through an evolutionarily conserved mechanism of pathway transducer de-ubiquitination, which might ultimately lead to dramatic wing hypoplasia and dysmorphia.

As already known, while the proteasome is a critical regulator of protein degradation, the destruction of organelles, RNA and long-lived proteins occurs via another cellular mechanism, called autophagy. Since not only autophagy, as an assembled machinery, but also *Atg* genes and their regulators are conserved from yeast to mammals, *Drosophila* represents a powerful model system to study autophagy *in vivo* [54]. Autophagy is essential in normal cell homeostasis and development, while its deregulation has been causally linked to a variety of human pathogenic states including cancer, neurodegenerative diseases and muscular disorders [55]. The protective role of autophagy against these maladies is mainly implemented through clearance of toxic cellular aggregates [56], [57]. In the present study, all transgenic fly eyes carrying reduced Atg (1, 4, 7, 9 and 18) protein contents presented with wild-type eye morphology and physiological ommatidium array, therefore indicating their (Atg) non-essential, or redundant, functional role during *Drosophila* eye development. Hence, autophagy might play a compensatory role, mainly as a protective mechanism, in fly eye homeostasis, only when other protein elimination systems, such as the proteasome particle, are inhibited, as previously reported in distinct biological settings [11], [58]–[60]. In *Drosophila*, it has been shown that induction of autophagy by overexpression of Atg1 largely suppresses Rheb-induced photoreceptor cell death as well as photoreceptor neural degeneration in models of Huntington’s disease [61]. Atg3 upregulation can also repress the rough eye phenotype caused by Dcp-1 overexpression [62]. Similarly, Atg7, when overexpressed, is able to fully rescue the rough eye phenotype developed by Hsp27 knockdown [63]. Our findings demonstrate that the RNAi-mediated disruption of autophagy-related gene expression produces flies with physiological eyes, therefore rendering autophagy a second line process likely controlling eye development only under stress conditions and not during its normal course. However, a redundant function of each Atg protein analyzed in autophagosome biogenesis, during fly eye formation, cannot be excluded.

Furthermore, we reveal that targeted downregulation of the same autophagy-related proteins (Atg1, 4, 7, 9 and 18) results in wings with -generally- physiological shape and size, even though loss of anterior cross-vein (cv-a) and/or ectopic vein tissue formation can be observed in all double transgenic fly populations examined. At the last step of wing metamorphosis in *Drosophila*, the wing epidermal cells are removed by programmed cell death during the wing spreading behavior after eclosion. Transmission electron microscopy reveals the association of this cell death type with extensive vacuole formation, thus indicating autophagy engagement [64]. The dying cells are detached from the wing cuticle and are absorbed into the thoracic cavity through wing veins. In flies that ectopically express p35 (an anti-apoptotic determinant) or a dominant-negative form of PKA (protein kinase A), cell death is inhibited, and adhesion of the dorsal and ventral cuticle is prevented, frequently causing blistered wings. Thus, the precise regulation of cell death is essential for morphogenesis of functional wings [64].

During fly wing development, Notch signaling activity is absolutely important for margin formation and also controls the refinement of broad proteins into the narrower veins observed in adults [26]. Loss of dAtg4 function enhances the phenotypes of Notch pathway mutants, indicating that downregulation of autophagy critically modulates Notch signaling capacity in *Drosophila* [65]. To this direction, the loss of anterior cross-vein and/or the ectopic vein formation in fly wings carrying reduced Atg (1, 4, 7, 9 and 18) protein contents, and thus compromised autophagy activity, could be tightly associated with a cell type-specific and position-dependent deregulation of Notch signaling function.

Conclusively, by applying the RNAi-based genetic technology to functionally eliminate numerous components and regulators of the two major proteolytic systems in a cell, proteasome and autophagy, we prove the fundamental role of proteasomal integrity and ubiquitin trafficking, and homeostasis, in fly eye and wing development. In contrast, autophagy presented to maintain a rather non-essential contribution in organ formation under physiological growth conditions. Of course, one cannot rule out a scenario of Atg protein redundancy that allows the uninterrupted biogenesis of autophagosomes, since the absence of one autophagic component could be successfully compensated by another. However, it is likely that upon exposure to stress autophagy may be activated, in an effort to detoxify the affected cells from poisonous proteins and organelles. Aging causes progressive damage of organs and tissues, and enhances their vulnerability to stress, while it also decreases the level of autophagic activity [23], [66]. Similarly, it has proved that pharmacological or genetic inhibition of autophagy induces degenerative changes in mammalian tissues that resemble those associated with aging [23], [67]. On the contrary, increased autophagy delays aging and even improves the cellular resistance to stress by enhancing the metabolic buffering capacity of cells and therefore expands lifespan (for detailed reviews see [23], [66]). In *Drosophila*, the expression of several autophagy-related genes is compromised by aging, while downregulation of autophagic genes results in the reduction of fly’s lifespan [68], [69]. In contrast, pharmacological or genetic induction of autophagy is able to significantly extend the lifespan of fruit fly [69]–[71]. Atg9 is a highly conserved multi-spanning membrane protein and is ubiquitously expressed in multi-cellular organisms. The cycling of Atg9 protein is essential for autophagosome formation [72], [73] and is also necessary for starvation-induced autophagy [74]. In this context and since increased metabolic demands and exercise activities (i.e. flying) have been shown to physiologically induce autophagy [75], it seems that downregulation of Atg9 renders aged wings vulnerable to the flying-induced metabolic and mechanical stress, therefore promoting the loss of wing’s -structural- integrity and resulting in strong organ’s fragility (broken wings). Given that autophagy is required for flying activity and is also compromised by aging, we herein prove that Atg9 represents an indispensable autophagic component of wing architecture and flying machinery in stressful environments, just like the ones developed by aging. Interestingly, ubiquitin/proteasome system (UPS) determinants seem to generally operate in a non-redundant fashion (possibly excluding the UbcD1 and 4 enzymes), as clearly demonstrated by the gene-specific dysmorphic phenotypes developed after their (UPS genes) RNAi-mediated downregulation in fly eye and wing discs.

By comparing the obtained β6_RNAi and β6^ts^ dysmorphic phenotypes, in both eye and wing organs, it proves that loss of a subunit is baneful for proteasome, whereas presence of a defective mutant component can be partly compensated by the whole assembly, thus implying a small degree of functional redundancy inside the proteasome particle. The more severe dysmorphic features developed in β2^ts^ and β6^ts^ triple transgenic eyes, compared to β2^ts^ or β6^ts^ double respective ones, strongly indicate that subunits of -at least- the same ring (i.e. “β”) carry not only distinct but also redundant activities, in an effort to ensure efficient proteasome function even in harsh cellular environments.

Further exploration of the importance of proteasome and autophagy integrity in developing cells undergoing stress will illuminate the “cross-talk” of the two major proteolytic machineries and allow the discovery of novel signaling mediators that critically control invertebrate eye and wing organ patho-physiology.
